# News and Events of the Week

**Published:** 1910-11-19

**Authors:** 


### News and Events of the Week.

The following meeting in connection with the League of Mercy will be held on Friday, November 25 :—Mid-Essex District : Mrs. Sheffield Neave, "At Home," Mill Green Park, Ingatestone, 3.30 p.m.

The International Cancer Conference, which recently met in Paris, has decided to meet next year at Dresden. It was also resolved that a meeting should be held in Brussels in 1913.

Sir Constantine Holman, M.D., of 26 Gloucester Place, Portman Square, W., who died at Ramsgate on August 18 last, aged eighty-one, left estate valued at £19,293 gross, with net personalty £18,423.

As a token of recognition of his untiring devotion to them while in hospital, during a recent severe epidemic of scarlet fever, the children of Prestbury, Lancashire, have presented Dr. J. H. Marsh, medical officer of health for Macclesfield, with a silver-mounted walking-stick and leather letter-case. At the same time Mrs. Marsh was given a hand-bag and a basket of flowers.

Mr. Norman MacDonald, of Casterton (Vic.), who has attained to the degree of Bachelor of Veterinary Science, has also been awarded a Science Research Scholarship. This is the first such scholarship that has been conferred by the Faculty of Science of the University of Melbourne upon a veterinary student. Mr. MacDonald will pursue his research work at the new Veterinary Research Institute in connection with the University.

Some months ago a mosquito-proof steamer was built to the order of a Liverpool firm for service on the West Coast of Africa. This has been followed by the construction of a cholera boat for the Russian Government. The latter vessel, launched at Helsingfors by the agents of Messrs. John I. Thornycroft, is primarily intended for the use of the medical officer in the Wilna district, and will be employed in transferring patients suffering from cholera to the hospitals.

At the International Food Congress which was held in Paris in 1909 there were many discussions on the subject of what should constitute purity in cocoa and chocolates, but the findings of the Congress were not universally accepted. Consequently a strong desire has arisen for a special Congress in connection with the subject, and it has been arranged that such should take place in Paris in January 1911, when there will be present delegates from Germany, England, Austria, Hungary, Belgium, Brazil, Spain, the United States, France, Holland, Italy, Mexico, and Switzerland. The Congress will be limited to five representatives from each nation, but each nation will only have the right to one vote, so that the delegates must arrange beforehand how their votes will be given. The Congress is being arranged under the auspices of the White Cross Society of Geneva, whose delegate in the United Kingdom is Mr. Loudon M. Douglas, F.R.S.E., of 3 Lauder Road, Edinburgh.

Lady Beachcroft appeals on behalf of the Poor Children's Yule-tide Association, whose aim is to provide Christmas gifts for as many as possible of the very poor children of the slums. Last year 134 Christmas-trees and 46,846 toys were sent to gladden the hearts of these gutter urchins, many of whom barely know the meaning of Christmas. To enable the Association to carry out its earnest wish materially to increase the number of recipients of its gifts, very generous help and support will be necessary. Subscriptions to the Poor Children's Yule-tide Association should be sent to the Hon. Secretary, at 32 John Street, Theobald's Road, W.C.

A clinic will be opened on January 1, 1911, in Garratt Lane, Wandsworth, which will be under the control of this division of the British Medical Association. It has received the approval of the London County Council and the Board of Education. At this clinic will be treated those children found on medical inspection to be defective in ears, nose, throat, or eyes, whose parents cannot afford the fees of the local doctors. The clinic will be used only by the children attending the L.C.C. schools within the L.C.C. Wandsworth Electoral area (excluding Streatham). A nurse, appointed by the L.C.C., will be in daily attendance. There will be a staff of seven local registered medical practitioners, who will hold office for only one year, and will be succeeded by others in rotation. They will attend during the afternoons. Cases of ringworm will be treated at the surgeries of local medical practitioners. The London County Council will elect the staff. A meeting will be held at the Town Hall, Wandsworth, on Friday, December 2, at 8 p.m., to discuss points affecting the treatment of the children in order that by the co-operation of all interested the clinic may prove successful. Suggestions for subjects for discussion should be sent to the Hon. Secretary, School Children Committee, Torquay House, Southfields, S.W., on or before Saturday, November 19. Resolutions based on the suggestions received will be drafted and submitted to the meeting. Invitations to attend are being issued by the Wandsworth Division of the British Medical Association to all registered medical practitioners resident and practising in the area defined, as well as to all members of the Care Committees and the heads of departments of the schools within that area.

### THE MEDICAL SCHOOLS.

The following are the arrangements for the week at the Central London Throat and Ear Hospital, Gray's Inn Road, W.C. : Lectures—Tuesday, November 22, at 3.45 p.m., anæsthetics, Dr. Kingsford; Friday, November 25, at 3.45 p.m., mouth and teeth.

The following are the arrangements for the week at the Medical Graduates' College and Polyclinic, 22 Chenies Street, W.C. : Monday, November 21, at 4 p.m., Dr. H. G. Adamson, Clinique (skin); at 5.15 p.m., Mr. T. Crisp English, "The Diagnosis and Treatment of Chronic Intestinal Obstruction." Tuesday, November 22, at 4 p.m., Dr. C. Theodore Williams, Clinique (medical); at 5.15 p.m.,

Dr. J. L. Bunch, "Some Methods of Treating Skin Diseases." Wednesday, November 23, at 4 P.M., Mr. T. P. Legg, Clinique (surgical); at 5.15 P.M., Dr. Arthur Latham, "The Administration and Value of Tuberculin in Various Forms of Tuberculosis." Thursday, November 24, at 4 P.M., Dr. E. Cautley, Clinique (medical); at 5.15 P.M., Dr. Arthur Latham, "The Administration and Value of Tuberculin in Various Forms of Tuberculosis." Friday, November 25, at 4 P.M., Mr. W. Stuart-Low, Clinique (ear, nose, and throat).

The following are the arrangements for next week at the West London Post-graduate College: November 21 and 24, demonstration by Surgical Registrar, at 10 A.M.; November 25, demonstrations by Medical Registrar, at 10 A.M.; November 22, demonstration of minor operations, at 11.30 A.M.; November 21, pathological demonstration, at 12 noon; November 22, lecture, Dr. Grainger Stewart, practical medicine, at 12.15 P.M. Lectures at 5 P.M.: November 21, Mr. Edwards on "Urinary Surgery"; November 22, Dr. Low on the "Prevention of Tropical Diseases"; November 23, Dr. Beddard on "Practical Medicine"; November 24, Dr. R. H. Cole on "The Modern Treatment of the Insane"; November 25, Dr. Abraham on "Cases of Skin Disease."

